# Patterns in mortality among people with severe mental disorders across birth cohorts: a register-based study of Denmark and Finland in 1982–2006

**DOI:** 10.1186/1471-2458-13-834

**Published:** 2013-09-11

**Authors:** Mika Gissler, Thomas Munk Laursen, Urban Ösby, Merete Nordentoft, Kristian Wahlbeck

**Affiliations:** 1 Nordic Research Academy in Mental Health, Nordic School of Public Health, Gothenburg, Sweden; 2 THL National Institute for Health and Welfare, Helsinki, Finland; 3 National Centre for Register-based Research, Aarhus University, Aarhus, Denmark; 4 Department of Psychiatry, Tiohundra AB, Norrtälje, Sweden; 5 Department of Molecular Medicine and Surgery, Karolinska Institutet, Stockholm, Sweden; 6 Psychiatric Centre Bispebjerg, Copenhagen University, Faculty of Health Sciences, Copenhagen, Denmark

**Keywords:** Birth cohort, Mental disorder, Mortality, Psychiatric care, Register study

## Abstract

**Background:**

Mortality among patients with mental disorders is higher than in general population. By using national longitudinal registers, we studied mortality changes and excess mortality across birth cohorts among people with severe mental disorders in Denmark and Finland.

**Methods:**

A cohort of all patients admitted with a psychiatric disorder in 1982–2006 was followed until death or 31 December 2006. Total mortality rates were calculated for five-year birth cohorts from 1918–1922 until 1983–1987 for people with mental disorder and compared to the mortality rates among the general population.

**Results:**

Mortality among patients with severe mental disorders declined, but patients with mental disorders had a higher mortality than general population in all birth cohorts in both countries. We observed two exceptions to the declining mortality differences. First, the excess mortality stagnated among Finnish men born in 1963–1987, and remained five to six times higher than at ages 15–24 years in general. Second, the excess mortality stagnated for Danish and Finnish women born in 1933–1957, and remained six-fold in Denmark and Finland at ages 45–49 years and seven-fold in Denmark at ages 40–44 years compared to general population.

**Conclusions:**

The mortality gap between people with severe mental disorders and the general population decreased, but there was no improvement for young Finnish men with mental disorders. The Finnish recession in the early 1990s may have adversely affected mortality of adolescent and young adult men with mental disorders. Among women born 1933–1957, the lack of improvement may reflect adverse effects of the era of extensive hospitalisation of people with mental disorders in both countries.

## Background

Life span has increased during the last decades in Europe. Since 1982, the life expectancy at birth increased by four years in Denmark and by six years in Finland [1]. Our previous studies have shown that the life expectancy among people with severe mental disorders also has increased from the 1980s in Denmark and Finland [2,3]. It is not clear whether this progress has been gradual across generations, or whether there are some birth cohorts who divert from the general picture. Even though the general trend has been positive, men with severe mental disorders still live 20 years less and women 15 years less than general population in the Nordic countries.

The excess mortality among people with severe mental disorders is not only caused by an increased risk for suicides and unintentional injuries, but also from an increased risk for mortality from diseases and medical conditions, such as diseases of the circulatory system, cancer and diabetes [4-6]. The literature suggests that this can partly be explained by low socioeconomic status [7,8], unhealthy lifestyle habits [9,10] and lack of access to health care with good quality [5,11]. Neither can the metabolic side effects of psychiatric medication in form of hyperglycemia and diabetes, weight gain, and lipid disturbances be excluded.

Both Denmark and Finland have undergone the major shift from an emphasis on psychiatric hospitalisations to integrated community-based mental health services. Between 1982 and 2006, the number of hospital beds in psychiatric hospitals per 100 000 population decreased from 171 to 63 in Denmark (−63%) and from 390 to 92 in Finland (−76%) [1]. The reduction reflects shorter treatment periods, improved primary health care based services and housing services (in Finland) and community mental health services (in Denmark), and the transfer of long-term inpatients in other institutions.

There are good possibilities for population-based studies on mortality among psychiatric patients in the Nordic countries, since the entire population is covered in the comprehensive nation-wide registers on general population, inpatient care and causes of death [6]. Mortality patterns are linked to macroeconomics, and increase in unemployment has been linked to higher suicide and alcohol-related mortality [12]. Economic recessions and depressions have been linked to increased risk of depression and anxiety as well as increased violent behaviour and excess use of alcohol and drugs, which have been hypothesised to have their origin in work-related stress and difficulties in family economy [13]. Excluding suicides, however, no data exists on the links between macroeconomics and mortality in the vulnerable group of people with severe mental disorders.

**Figure 1:**
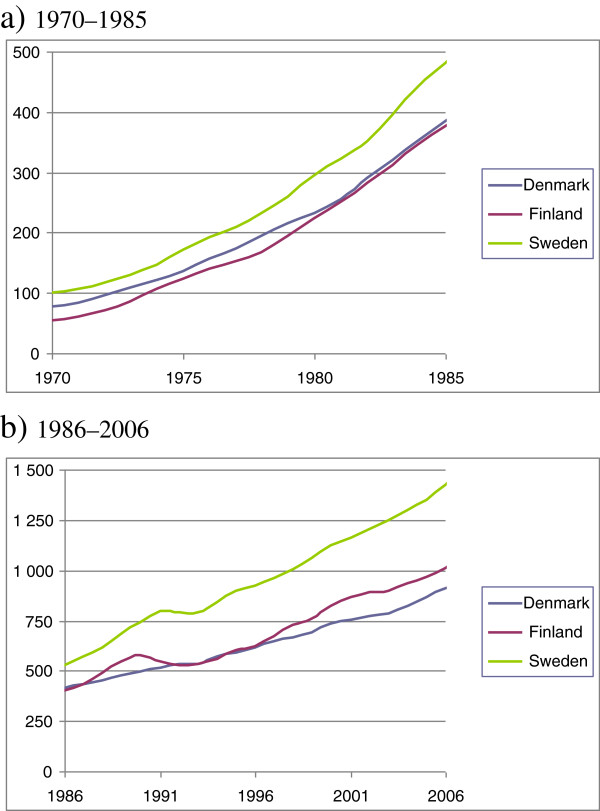
**Gross Domestic Product (GDP) in Denmark, Finland and Sweden in a) 1970–1985 and b) 1986–2006.** The GDP in Sweden in 1970 (22 772 Swedish Crowns, 4402 US Dollars) is the reference point (1970 = 100).

Denmark and Finland are Nordic countries with a similar culture, societal structure and welfare system. In spite of the social and cultural similarities, they differ in macroeconomic trends due to differing trade and industry. Denmark had a slower economic growth than other Nordic countries in the 1970s (Figure 1), and the country faced a recession 1980s with unemployment rates between 8% and 10%. The unemployment rose also in the beginning of the 1990s up to 12%, but cannot be compared to the Finnish rates in the early 1990s. Finland experienced then a sudden and severe economic recession with a five-fold increase in unemployment and a decrease of more than 10% in the GDP, which led to cuts in public services. The GDP remained below the level of 1990 for five years until 1995 (Figure 1).

Our aim was to compare different birth cohorts to investigate if the development in relative mortality among people with mental disorders in birth cohorts at risk were similar in the two study countries Denmark and Finland, and especially whether major macro-economic cycles had an impact on these trends. A specific question was whether stagnating or declining economy affected the mortality among people with severe mental disorders leading to hospitalisation. Any significant differences may be caused by differences how the contemporary societal changes have affected cultural generations, i.e. cohorts of people who were born in the same year range and share similar socio-cultural experience.

## Methods

### Case definition

Cases of severe mental disorders included were identified from discharge diagnoses recorded in the nationwide hospital discharge registers. Both countries use the International Classification of Diseases (ICD), established by the World Health Organization (WHO), for definition and classification of psychiatric and physical diseases in their hospital discharge registers. We used the primary diagnoses given in ICD-8, ICD-9 or ICD-10, recorded for each hospitalisation to define our study population with diagnosed mental disorder. The diagnoses given in ICD-8 and ICD-9 were transformed to ICD-10 diagnoses.

All patients admitted at least once during the period 1 January 1982 and 31 December 2006 with a primary diagnosis of mental disorder (ICD-10: F10-F69) were retrieved from the Danish and Finnish national hospital registers. Patients with a diagnosis of intellectual disability (F70-79) at any point in time were excluded. Hospitalisations due to organic mental disorders, e.g. dementia, (F00-09) resulted in exclusion of the subject starting from the first hospitalisation due to dementia and any episode afterwards. Patients with a diagnosis related to intellectual disability and dementia were excluded because of the high risk for premature mortality inherent to the organic nature of these disorders.

### Information on deaths

Information on deaths was taken from national cause-of-death registers, which cover all citizens and permanent residents, and linked to the hospital data with the unique personal identity code, which is given to all citizens at birth and permanent residents at migration.

### Data sources

#### Denmark

The Danish Psychiatric Central Register [14] covers all psychiatric inpatient facilities in Denmark and has been computerised since 1969. In Denmark, the ICD-8 classification was used as the diagnostic system used until 1993 and the ICD-10 was introduced in 1994.

The Danish Cause of Death Register contains information about all deaths of Danish citizens and residents, date of death, and circumstances and causes of death. The register has a high level of completeness and its validity has been evaluated with very good results [15].

#### Finland

The Finnish Hospital Discharge Register (FHDR) includes data on all inpatient episodes on an individual level since 1969. For diagnosis, ICD-8 was used during the period 1969–1986, ICD-9 during the period 1987–1995 and ICD-10 from 1996 onwards. The FHDR has been found to be a valid and reliable tool for epidemiological research [16].

The Finnish Cause of Death Register records data on the deaths of all citizens and permanent residents in Finland. The register has a high level of completeness. All diagnoses of the causes of death have to pass a routine validation carried out by regional medical officers and physicians at Statistics Finland. Generally, the quality has been found to be very good [17].

### Statistical analysis

The population at risk consisted of all patients admitted at least once during the period 1 January 1982 and 31 December 2006. Mortality follow-up was based on death during the same period. The mortality rates were studied separately for men and women for five-year birth cohorts born in 1918–1922 until 1983–1987 and for death year groups, grouped in five year periods as follows: 1982–1986, 1987–1991, 1992–1996, 1997–2001 and 2002–2006. Basic information on the number of cases and deaths are presented in Table 1.

**Table 1:** The number of follow-up years and deaths by study period, birth cohort and sex in Denmark and Finland

	**Men**						**Women**					
	**1982-86**	**1987-91**	**1992-96**	**1997-01**	**2002-06**	**Total**	**1982-86**	**1987-91**	**1992-96**	**1997-01**	**2002-06**	**Total**
Finland												
Follow-up years												
Total	177 301	276 855	493 517	630 559	733 859	2 312 091	115 501	181 663	330 736	447 783	559 779	1 635 462
1918-22	7 273	8 214	10 255	8 614	5 773	40 129	8 885	11 898	18 584	20 436	18 710	78 513
1923-27	11 723	13 625	18 069	16 609	13 342	73 368	10 518	14 379	22 582	26 055	26 876	100 410
1928-32	14 935	19 009	26 821	26 314	23 159	110 238	11 832	16 302	25 689	29 873	32 106	115 802
1933-37	15 997	21 384	32 702	34 651	33 223	137 957	11 324	17 068	27 671	31 371	33 571	121 005
1938-42	18 685	26 567	42 663	47 591	47 406	182 912	12 021	18 135	31 437	38 014	41 441	141 048
1943-47	24 691	35 961	60 102	70 736	74 320	265 810	15 123	22 867	40 130	51 227	58 160	187 507
1948-52	27 652	41 733	71 882	86 822	94 882	322 971	16 335	24 960	44 048	57 181	68 249	210 773
1953-57	22 840	35 087	62 030	77 920	88 976	286 853	13 567	21 503	38 915	51 502	62 540	188 027
1958-62	18 598	27 723	50 089	64 249	75 635	236 294	8 694	15 107	29 397	41 345	52 745	147 288
1963-67	13 707	31 620	54 201	67 333	78 129	244 990	5 809	11 821	24 501	35 897	46 752	124 780
1968-72	1 200	14 906	45 944	58 408	67 868	188 326	1 393	6 380	16 466	26 145	35 879	86 263
1973-77		1 024	17 079	43 817	56 343	118 263		1 243	9 021	19 421	29 536	59 221
1978-82			1 680	24 704	53 674	80 058			2 295	14 747	29 229	46 271
1983-87				2 791	21 129	23 920				4 569	23 985	28 554
Deaths												
Total	4 266	7 347	12 633	16 289	18 854	59 389	1 584	2 781	5 207	7 797	10 022	27 391
1918-22	495	722	1 148	1 153	910	4 428	298	588	1 200	1 711	1 862	5 659
1923-27	644	935	1 509	1 657	1 464	6 209	288	481	896	1 398	1 737	4 800
1928-32	570	984	1 626	1 822	1 947	6 949	216	384	646	929	1 337	3 512
1933-37	497	811	1 341	1 663	1 880	6 192	159	260	477	740	870	2 506
1938-42	472	794	1 328	1 803	2 029	6 426	144	266	427	630	799	2 266
1943-47	510	891	1 561	2 101	2 633	7 696	155	232	441	648	909	2 385
1948-52	457	890	1 574	2 158	2 772	7 851	121	240	405	627	842	2 235
1953-57	328	637	1 068	1 526	1 902	5 461	116	155	308	475	654	1 708
1958-62	201	343	640	984	1 275	3 443	68	82	192	260	397	999
1963-67	86	216	474	622	835	2 233	16	55	117	181	244	613
1968-72	6	113	270	409	489	1 287	3	36	64	92	131	326
1973-77		11	89	254	341	695		2	31	59	88	180
1978-82			5	132	280	417			3	40	90	133
1983-87				5	97	102				7	62	69
Denmark												
Follow-up years												
Total	90 515	46 033	36 595	39 291	43 803	256 237	91 650	48 089	39 826	44 004	47 647	271 216
1918-22	4 524	1 692	1 205	756	417	8 594	7 930	3 643	2 463	1 989	1 029	17 054
1923-27	5 675	2 073	1 340	919	564	10 571	9 059	3 818	2 562	2 149	1 475	19 063
1928-32	7 244	2 628	1 533	1 084	738	13 227	9 416	3 799	2 395	1 879	1 362	18 851
1933-37	8 610	3 296	1 940	1 312	1 076	16 234	10 215	4 361	2 588	2 002	1 543	20 709
1938-42	10 544	4 295	2 505	1 935	1 649	20 928	11 155	4 926	3 203	2 412	1 891	23 587
1943-47	14 555	6 243	3 942	3 215	2 672	30 627	13 439	6 466	4 262	3 600	2 813	30 580
1948-52	13 700	6 100	4 050	3 614	3 244	30 708	10 480	5 479	4 128	3 889	3 253	27 229
1953-57	11 866	6 013	4 637	4 241	3 888	30 645	8 978	4 777	4 146	4 018	3 821	25 740
1958-62	8 456	5 069	4 363	4 672	4 822	27 382	6 338	4 068	3 779	4 181	4 284	22 650
1963-67	4 729	5 329	4 314	5 028	5 738	25 138	4 015	3 598	3 567	4 420	4 827	20 427
1968-72	612	2 811	3 974	4 281	5 179	16 857	625	2 556	3 126	4 132	4 876	15 315
1973-77		484	2 309	4 697	5 034	12 524		598	2 869	4 502	5 039	13 008
1978-82			483	2 936	5 006	8 425			738	3 805	5 648	10 191
1983-87				601	3 776	4 377				1 026	5 786	6 812
Deaths												
Total	2 856	1 472	1 242	1 243	1 192	8 005	2 132	1 147	923	934	894	6 030
1918-22	425	187	181	147	96	1 036	392	225	207	194	130	1 148
1923-27	370	217	139	136	106	968	365	200	160	179	127	1 031
1928-32	372	161	107	99	98	837	290	158	134	100	124	806
1933-37	339	170	104	106	84	803	246	134	76	84	87	627
1938-42	305	154	121	108	109	797	232	128	85	67	83	595
1943-47	364	165	132	134	124	919	237	104	82	98	70	591
1948-52	290	122	119	118	141	790	164	71	54	64	73	426
1953-57	231	116	113	126	117	703	121	55	36	46	62	320
1958-62	127	71	79	85	88	450	62	36	37	41	55	231
1963-67	33	81	71	67	80	332	23	23	19	27	29	121
1968-72	0	27	47	56	53	183	0	11	22	15	22	70
1973-77		1	27	34	52	114		2	10	13	13	38
1978-82			2	25	33	60			1	6	11	18
1983-87				2	11	13				0	8	8

Comparisons were made for total mortality rate for the whole population for the same birth cohorts and for both sexes. Observed/expected ratios (O/E ratios) with 95% confidence intervals were calculated for each mortality rate comparison. Expected mortality rates were based on mortality rates among total population provided by sex and five-year age groups by the national statistical offices. The mortality differences between birth cohorts were calculated by using the test for relative proportions. The statistical analysis was made by using SAS version 9.3.

## Results

**Figure 2:**
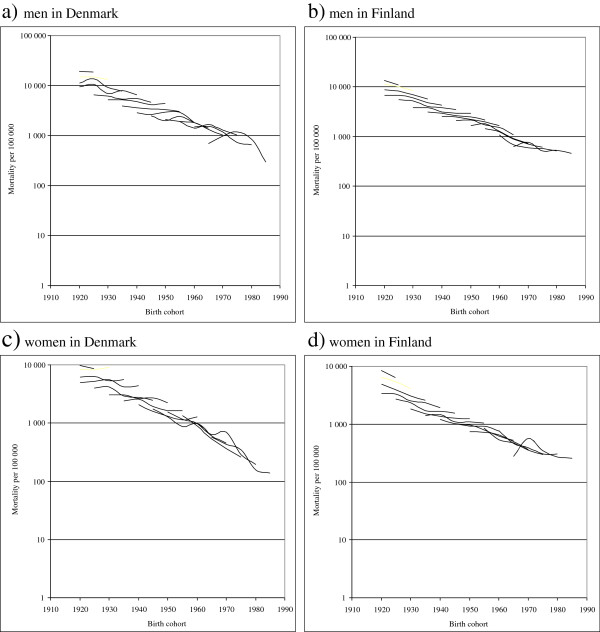
**Mortality with severe mental disorder by birth cohort in 1982–2006, logarithmic scale a) men in Denmark, b) men in Finland, c) women in Denmark, d) women in Finland.** Each line represents an age group through the follow-up period of up to 25 years.

Overall mortality, measured as total number of deaths per 100,000 years of follow-up, among patients with severe mental disorders declined for each cohort in both countries (Table 2, Figure 2). For Danish men aged 15–34 years old and women aged 15–39 years old as well as for Finnish men and women aged 20–34 years old, the mortality rates more than halved during the study period. The smallest decline was observed for Finnish men aged 15–19 years old (−27%) and 50–54 years old (−23%) as well as for Danish men aged 45–59 years old (−24%, -15% and −29% in each five-year age group, respectively). For women, the smallest decline was observed in Finland in age group 15–19 years old (−6%), and in Denmark in age groups 45–54 years old (−26% and −27% in the two five-year age groups, respectively).

**Table 2:** Mortality per 100 000 among women with severe mental disorders requiring hospitalization by birth cohort in 1982-2006

**Men**													
Denmark													
	15-19	20-24	25-29	30-34	35-39	40-44	45-49	50-54	55-59	60-64	65-69	70-74	75-79
1918-22										9 394	11 050	15 023	19 452
1923-27									6 520	10 470	13 706	14 801	18 793
1928-32								5 136	6 125	6 981	9 131	13 275	
1933-37							3 937	5 158	5 361	8 078	7 806		
1938-42						2 893	3 586	4 831	5 582	6 612			
1943-47					2 501	2 643	3 349	4 168	4 641				
1948-52				2 117	2 000	2 938	3 265	4 346					
1953-57			1 947	1 929	2 437	2 971	3 009						
1958-62		1 502	1 401	1 811	1 819	1 825							
1963-67	698	1 520	1 646	1 333	1 394								
1968-72	961	1 183	1 308	1 023									
1973-77	1 170	724	1 033										
1978-82	852	659											
1983-87	291												
Finland													
1918-22										6 806	8 790	11 195	13 385
1923-27									5 493	6 862	8 351	9 977	10 973
1928-32								3 817	5 176	6 062	6 924	8 407	
1933-37							3 107	3 793	4 101	4 799	5 659		
1938-42						2 526	2 989	3 113	3 789	4 280			
1943-47					2 066	2 478	2 597	2 970	3 543				
1948-52				1 653	2 133	2 190	2 486	2 922					
1953-57			1 436	1 815	1 722	1 958	2 138						
1958-62		1 081	1 237	1 278	1 532	1 686							
1963-67	627	683	875	924	1 069								
1968-72	758	588	700	721									
1973-77	521	580	605										
1978-82	534	522											
1983-87	459												
Women													
Denmark													
	15-19	20-24	25-29	30-34	35-39	40-44	45-49	50-54	55-59	60-64	65-69	70-74	75-79
1918-22										4 943	6 176	8 405	9 756
1923-27									4 029	5 239	6 244	8 329	8 610
1928-32								3 080	4 159	5 596	5 323	9 107	
1933-37							2 408	3 073	2 937	4 197	5 637		
1938-42						2 080	2 599	2 654	2 778	4 390			
1943-47					1 764	1 609	1 924	2 722	2 489				
1948-52				1 565	1 296	1 308	1 646	2 244					
1953-57			1 348	1 151	868	1 145	1 622						
1958-62		978	885	979	981	1 284							
1963-67	573	639	533	611	601								
1968-72	430	704	363	451									
1973-77	349	289	258										
1978-82	158	195											
1983-87	138												
Finland													
1918-22										3 354	4 942	6 457	8 372
1923-27									2 738	3 345	3 968	5 366	6 463
1928-32								1 826	2 356	2 515	3 110	4 164	
1933-37							1 404	1 523	1 724	2 359	2 592		
1938-42						1 198	1 467	1 358	1 657	1 928			
1943-47					1 025	1 015	1 099	1 265	1 563				
1948-52				741	962	919	1 097	1 234					
1953-57			855	721	791	922	1 046						
1958-62		782	543	653	629	753							
1963-67	275	465	478	504	522								
1968-72	564	389	352	365									
1973-77	344	304	298										
1978-82	271	308											
1983-87	258												

In both countries and in all cohorts, patient with severe mental disorders had a higher mortality than general population (Table 3 for men and Table 4 for women). Generally, the excess mortality was higher in Denmark than in Finland. Among Danish men aged 15–64 years old, the mean excess mortality was 9-fold in 1982–86, but declined to 7-fold in 2002–2006 compared to general population (p < 0.001). For Finnish men, the excess mortality remained between 4- and 5-fold during the whole study period. For women in the same age groups, the mean excess mortality declined in both countries. The relative improvement was larger for Danish women (from 12-fold in 1982–86 to 6-fold mortality in 2002–06, p < 0.001) than for Finnish women (from 9-fold to 6-fold, p < 0.001). By age groups, the excess mortality declined most for Danish men and women aged 15–49 years old as well as for Finnish men aged 25–39 years old and Finnish women aged 20–59 years old.

**Table 3:** Excess mortality calculated as observed/expected ratio with 95% confidence intervals among male patients with mental disorders compared with general population, by birth cohort, Denmark and Finland 1982-2006

	**1982-86**	**1987-91**	**1992-96**	**1997-01**	**2002-06**
Denmark					
1918-22	3.8 (3.2-4.6)	3.0 (2.6-3.4)	2.7 (2.4-3.0)	2.4 (2.2-2.7)	2.0 (1.8-2.2)
1923-27	6.8 (6.3-7.4)	7.2 (5.4-9.4)	6.2 (5.0-7.5)	4.7 (4.0-5.5)	4.3 (3.8-4.8)
1928-32	5.4 (4.8-6.0)	4.2 (2.7-5.7)	3.1 (2.5-3.8)	2.9 (2.7-3.0)	3.0 (2.6-3.5)
1933-37	6.9 (6.3-7.6)	5.7 (4.5-6.9)	4.0 (3.9-4.2)	4.3 (4.1-4.4)	3.0 (2.6-3.4)
1938-42	8.9 (8.1-9.7)	7.0 (6.1-7.9)	6.3 (6.1-6.4)	4.9 (4.7-5.2)	4.1 (3.8-4.4)
1943-47	11.8 (10.8-12.9)	8.0 (7.4-8.5)	6.9 (6.5-7.2)	5.9 (5.6-6.2)	4.5 (4.4-4.7)
1948-52	12.5 (11.4-13.7)	8.9 (8.3-9.5)	9.0 (8.5-9.5)	7.1 (6.7-7.5)	6.4 (6.1-6.8)
1953-57	15.2 (13.6-16.9)	11.8 (10.9-12.7)	10.2 (9.6-10.9)	9.7 (9.2-10.2)	6.7 (6.2-7.2)
1958-62	13.5 (11.7-15.5)	11.5 (10.3-12.8)	11.8 (10.9-12.8)	10.0 (9.3-10.7)	6.9 (6.3-7.4)
1963-67	8.2 (6.5-10.1)	14.3 (12.5-16.3)	14.9 (13.5-16.3)	10.8 (10.0-11.6)	8.6 (8.1-9.2)
1968-72		12.7 (10.5-15.3)	13.7 (12.1-15.5)	14.1 (12.8-15.5)	9.6 (8.8-10.5)
1973-77			16.6 (13.3-20.4)	10.1 (8.9-11.4)	12.4 (11.1-13.8)
1978-82				12.0 (10.1-14.3)	8.6 (7.6-9.7)
1983-87					4.3 (3.5-5.2)
Finland					
1918-22	1.2 (1.1-1.3)	2.2 (2.0-2.5)	2.0 (1.9-2.2)	1.7 (1.6-1.8)	1.4 (1.3-1.5)
1923-27	3.6 (3.3-3.9)	4.3 (3.4-5.4)	3.9 (3.3-4.5)	3.4 (3.0-3.8)	2.7 (2.5-3.0)
1928-32	2.5 (2.2-2.7)	3.3 (2.5-4.0)	2.8 (2.3-3.3)	2.4 (2.3-2.5)	2.1 (1.9-2.3)
1933-37	2.6 (2.4-2.9)	3.9 (3.3-4.6)	3.2 (2.8-3.6)	2.7 (2.6-2.9)	2.4 (2.1-2.6)
1938-42	4.0 (3.7-4.4)	4.7 (4.1-5.2)	3.7 (3.4-4.1)	3.3 (3.1-3.5)	2.7 (2.6-2.9)
1943-47	5.9 (5.4-6.4)	5.7 (5.3-6.1)	4.6 (4.4-4.9)	3.8 (3.7-4.0)	3.3 (3.2-3.4)
1948-52	6.3 (5.7-6.9)	6.7 (6.2-7.2)	5.2 (4.9-5.6)	4.7 (4.4-4.9)	3.9 (3.6-4.1)
1953-57	6.1 (5.5-6.8)	8.0 (7.4-8.6)	6.3 (5.8-6.7)	5.4 (5.1-5.7)	4.3 (4.0-4.6)
1958-62	6.2 (5.4-7.1)	7.6 (6.8-8.5)	7.2 (6.6-7.8)	6.6 (6.2-7.0)	5.3 (5.0-5.6)
1963-67	4.8 (3.9-5.9)	5.0 (4.4-5.8)	6.1 (5.5-6.7)	6.0 (5.6-6.5)	5.4 (5.0-5.8)
1968-72		6.0 (5.0-7.3)	5.0 (4.5-5.7)	5.5 (5.0-6.1)	5.4 (4.9-5.9)
1973-77			5.4 (4.4-6.7)	5.3 (4.7-6.0)	5.5 (5.0-6.2)
1978-82				6.0 (5.0-7.1)	5.0 (4.4-5.6)
1983-87					5.5(4.4-6.7)

**Table 4:** Excess mortality calculated as observed/expected ratio with 95% confidence intervals among female patients with mental disorders compared with general population, by birth cohort, Denmark and Finland 1982-2006

	**1982-86**	**1987-91**	**1992-96**	**1997-01**	**2002-06**
Denmark					
1918-22	3.7 (3.0-4.6)	3.0 (2.6-3.5)	2.6 (2.4-2.7)	2.0 (1.8-2.1)	1.6 (1.5-1.7)
1923-27	6.5 (5.8-7.3)	5.6 (2.6-9.9)	4.4 (3.2-5.9)	4.0 (3.1-5.0)	2.9 (2.4-3.5)
1928-32	5.0 (4.4-5.7)	4.5 (4.0-5.0)	4.0 (3.6-4.3)	2.5 (1.8-3.2)	3.1 (2.7-3.5)
1933-37	6.3 (5.4-7.4)	5.1 (4.5-5.8)	3.3 (3.0-3.7)	3.2 (2.7-3.8)	3.2 (3.0-3.4)
1938-42	9.2 (7.7-10.8)	7.3 (6.4-8.2)	5.1 (4.6-5.6)	3.7 (3.4-4.0)	4.3 (4.0-4.7)
1943-47	13.1 (11.1-15.3)	7.5 (6.6-8.5)	5.8 (5.3-6.4)	5.8 (5.4-6.3)	3.7 (3.2-4.3)
1948-52	18.0 (15.0-21.6)	10.3 (9.0-11.6)	6.1 (5.5-6.8)	5.5 (5.0-5.9)	5.2 (4.7-5.7)
1953-57	23.9 (19.8-28.7)	14.8 (12.6-17.3)	6.7 (6.0-7.5)	6.2 (5.6-6.7)	5.9 (5.5-6.4)
1958-62	24.4 (18.9-30.9)	17.3 (13.7-21.5)	13.3 (11.5-15.3)	9.0 (7.9-10.1)	8.3 (7.5-9.1)
1963-67	18.2 (10.4-29.5)	17.9 (13.5-23.4)	12.6 (10.4-15.1)	9.8 (8.4-11.4)	6.7 (5.9-7.6)
1968-72		14.7 (10.3-20.3)	22.9 (17.6-29.3)	9.4 (7.6-11.5)	8.8 (7.4-10.4)
1973-77			12.2 (8.3-17.3)	10.4 (7.9-13.4)	8.1 (6.5-10.0)
1978-82				6.8 (4.9-9.3)	7.1 (5.7-8.8)
1983-87					6.3 (4.8-8.0)
Finland					
1918-22	2.1 (1.8-2.5)	2.8 (2.5-3.3)	2.3 (2.1-2.5)	1.8 (1.6-2.0)	1.3 (1.1-1.5)
1923-27	6.6 (5.8-7.4)	5.8 (3.1-9.2)	4.8 (3.4-6.5)	4.3 (3.4-5.4)	3.3 (2.8-3.9)
1928-32	4.4 (3.8-5.0)	4.1 (3.7-4.5)	3.0 (2.8-3.3)	2.5 (1.7-3.3)	2.2 (1.8-2.5)
1933-37	5.8 (4.9-6.7)	4.2 (3.7-4.7)	3.4 (3.1-3.8)	3.2 (2.6-3.8)	2.5 (2.3-2.6)
1938-42	6.9 (5.8-8.1)	5.7 (5.0-6.4)	4.0 (3.6-4.4)	3.6 (3.3-3.9)	2.9 (2.6-3.1)
1943-47	11.3 (9.6-13.2)	6.5 (5.7-7.4)	4.7 (4.3-5.2)	3.9 (3.6-4.2)	3.4 (3.0-3.7)
1948-52	11.4 (9.4-13.6)	8.5 (7.5-9.7)	5.9 (5.3-6.5)	4.6 (4.3-5.0)	3.8 (3.4-4.1)
1953-57	17.2 (14.2-20.6)	10.0 (8.5-11.7)	7.7 (6.8-8.6)	6.1 (5.6-6.7)	4.7 (4.3-5.0)
1958-62	22.0 (17.1-27.9)	11.6 (9.2-14.4)	10.0 (8.6-11.5)	6.7 (5.9-7.6)	5.3 (4.8-5.9)
1963-67	8.2 (4.7-13.3)	12.0 (9.0-15.6)	10.5 (8.7-12.6)	7.8 (6.7-9.0)	6.5 (5.7-7.4)
1968-72		13.9 (9.8-19.3)	10.8 (8.3-13.8)	8.9 (7.2-10.9)	6.6 (5.5-7.8)
1973-77			11.5 (7.8-16.3)	8.7 (6.7-11.3)	8.5 (6.8-10.5)
1978-82				8.7 (6.2-11.8)	9.2 (7.4-11.3)
1983-87					8.9 (6.8-11.4)

Two exceptions were observed when investigating mortality by birth cohorts. First, the excess mortality risk stagnated among Finnish men born in 1963–1987, and remained more than five-fold compared to general population at ages 15–24 years. In both countries, the mortality among male patients with severe mental disorders decreased, when comparing men born 1963–72 and 1978–87 (<0.001), but the decline was more substantial in Denmark (−43%) compared to Finland (−23%). The excess mortality declined in Denmark from being 11.9-fold (95% confidence interval 10.0-14.0) for men born 1963–72 to 8.7-fold (7.1-10.5) for men born 1978–87, while no progress was observed in Finland: the excess risks for mortality among patients with severe mental disorders were 5.2-fold (4.7-5.8) for Finnish men born 1978–82 and 5.3-fold (4.9-5.7) for Finnish men born 1983–87.

Second, the excess mortality risk stagnated for Danish and Finnish women born in 1933–1957 at their 40s. Their mortality remained six-fold in Denmark and Finland at ages 45–49 years and seven-fold in Denmark at ages 40–44 years. Between cohorts 1933–37 and 1953–57, the mortality at 40–44 years and 45–49 years declined both in Denmark (−33%, p = 0.002 and −27%, p < 0.001) and in Finland (−26%, p < 0.001 and −19%, p < 0.001), but the excess mortality remained at the same level. In Denmark, it was for women aged 40–44 years old 6.3-fold (95% confidence interval 6.1-6.5) for women born 1933–37 and 5.9-fold (5.5-6.3) for women born 1953–57. For women aged 45–49 years old the excess risks were 5.1-fold (4.9-5.4) and 5.2-fold (4.9-5.5), respectively. For Finland, the excess mortality decreased for women aged 40–44 years old from 5.8-fold (5.6-6.0) for women born 1933–37 to 4.7-fold (4.5-4.9) for women born 1953–57, but the difference remains statistically insignificant for women aged 45–49 years with a change from being 4.2-fold (4.0-4.4) to being 3.8-fold (3.6-4.0).

## Discussion

Our data confirmed the declining mortality trends among hospitalised patients with severe mental disorders in Denmark and Finland. In general, the mortality gap diminished for each consecutive birth cohort, but patients with mental disorders still had a significantly higher mortality rate than the total population in general.

We observed that the relative mortality among young Finnish men born in 1963–1987 with severe mental disorders leading to hospitalisation did not improve at all. One explanation may be the Finnish recession in the early 1990s, which seems to have affected these birth cohorts most. At the time of the recession in Finland, these boys and young men were affected by adversities in their families of origin [18] and faced considerable problems in accessing the labour market [19].

The sex-specific effect may be explained by socio-economic disparities, which significantly differ between men and women. The educational level of Finnish young men is lower than among young women. In 2009, 23% of men aged 25–29 years old and 34% of men aged 30–34 years old had a tertiary education, while the percentages were substantially higher (40% and 53%, respectively) for women in the same age groups [20]. Also the unemployment figures have been higher for men aged less than 25 years old. This suggests that the likelihood to be excluded or underprivileged has remained high among young Finnish men during the 1990s recession and after it.

Also women with severe mental disorders born before, during or after World War II, in the period 1933–1957, failed to reduce their excess mortality. This may reflect a generation of women with severe mental disorders who initially were extensively hospitalised due to their mental disorder, and were too old to benefit from the deinstitutionalised psychiatry which began to evolve in the 1970s. During the era of high level of psychiatric hospitalisation, people with mental disorders that nowadays are treated in community care were exposed to extensive hospitalisation periods, which resulted in iatrogenic adverse effects on level of functioning. It is possible that our findings illustrate a “lost generation”, i.e. a generation with excess mortality due to excess hospitalisation. Previous reports have indicated an excess mortality among in-patient psychiatric populations [21,22].

### Limitations

Our study data covered all institutionalised people with mental disorders in Denmark and Finland during 25 years. The data collection systems are obligatory and their quality for register-based research has been shown to be good [14,16]. Also, the same exclusion and inclusion criteria were applied for both countries. The register-based data have, however, its limitations.

There may be differences in the provision of health services, especially in the use of inpatient care services between the two study countries. The proportion of untreated or inappropriately treated people with severe mental disorder may differ in the two study countries and also during the study period. The distribution of diagnoses is different, since Denmark has reported more depression and drug-related treatments, while schizophrenia and alcohol-related treatments were more common in Finland [23]. Epidemiological studies confirm that schizophrenia spectrum disorders [24] and alcohol use disorders [25] may be more common in Finland than in other countries. Furthermore, our analyses do not allow complete comparisons between cohorts. For the older ones, the people with most serious mental disorders have already died, and thus they are excluded from our data.

The data was based on admission data, but the cohort definition was based on primary diagnoses at discharge, which is more accurate measure for patient with mental disorders than the admission diagnoses. Our data did not cover all psychiatric diagnoses. People with intellectual disabilities were excluded. The patients were also excluded from the date they received a diagnoses related to organic mental disorder, such as dementia. Both of these patient groups have high risk for premature mortality. Furthermore, we could not compare the distribution of mental disorders by year cohort due to differences in the register data in Denmark and Finland. Such differences by age group are well-known, but we cannot say, if these varied between the two study countries.

Our study data did not include information on international migration. Thus people who have permanently migrated abroad are included in the population at risk even though they may have died after leaving the country where they were treated. Since the migration rates are relatively low in the Nordic countries, we can estimate that the effect of not having information on migration is minor.

Due to the long follow-up period we were not able to get detailed background information on the people with severe mental disorders. The register-based information systems based on personal identification numbers in Denmark and Finland have been built from the 1970s onwards, and the information available before that is very limited. Therefore, we had to limit our analyses to basic variables available in the data sources, and thus, our conclusions remain partly speculative.

## Conclusions

Although our data indicate that for each birth cohort the mortality gap between people with mental disorders is decreasing, our results also indicate that the favourable overall trend in this vulnerable population can easily be offset by selective disadvantages. Two major societal changes, i.e. the deep Finnish recession in the 1990s and the excessive long-term hospitalisation of people with mental disorders in the 1950s to 1970s, may have contributed to lack of progress in equity in terms of mortality in groups who were particularly exposed to these major societal changes.

Besides accessible and responsive primary health care, active labour market policy, social welfare policies supporting families and parenting and programmes to support unmanageable dept should be used to diminish mental and somatic health problems during economic downturn and recession [26]. If the economic crisis continues for a longer time, it is important to fight against poverty and its inheritance, since mental health problems effect also families. [27].

## Competing interests

The authors have no competing interest to report.

## Authors’ contributions

MG, TML, MN and KW planned the study. MG and TML made the analyses. MG wrote the article with contributions from all other authors (TML, UÖ, MN and KW). All authors read and approved the final manuscript.

## Pre-publication history

The pre-publication history for this paper can be accessed here:

http://www.biomedcentral.com/1471-2458/13/834/prepub
